# A Study of Outliers in GNSS Clock Products

**DOI:** 10.3390/s24030799

**Published:** 2024-01-25

**Authors:** Kamil Maciuk, Inese Varna, Karolina Krzykowska-Piotrowska

**Affiliations:** 1 Department of Integrated Geodesy and Cartography, AGH University, Mickiewicza 30 Ave, 30-059 Kraków, Poland; 2 Institute of Geodesy and Geoinformatics, University of Latvia, Jelgavas 3, LV-1004 Riga, Latvia; inese.varna@lu.lv; 3 Faculty of Transport, Warsaw University of Technology, ul. Koszykowa 75, 00-662 Warsaw, Poland

**Keywords:** GNSS, clock, outlier, GPS, satellite

## Abstract

Time is an extremely important element in the field of GNSS positioning. In precise positioning with a single-centimetre accuracy, satellite clock corrections are used. In this article, the longest available data set of satellite clock corrections of four GNSS systems from 2014 to 2021 was analysed. This study covers the determination of the quality (outliers number and magnitude), availability, stability, and determination of the specificity and nature of the clock correction for each satellite system. One problem with the two newest satellite systems (Galileo and BeiDou) is the lack of availability of satellite signals in the early years of the analysis. These data were available only in the later years of the period covered by the analysis, as most of the satellites have only been in orbit since 2018–2019. Interestingly, the percentage of outlying observations was highest in Galileo and lowest in BeiDou. Phase and frequency plots showed a significant number of outlying observations. On the other hand, after eliminating outlying observations, each system showed a characteristic graph waveform. The most consistent and stable satellite clock corrections are provided by the GPS and GLONASS systems. The main problems discussed in this paper are the determination of the number and magnitude of outliers in clock products of four GNSS systems (GPS, GLONASS, Galileo, Beidou) and the study on the long-term stability of GNSS clocks analysis, which covers the years 2014–2021.

## 1. Introduction

For highly demanding applications such as geodesy, civil engineering or surveying, there is a general desire to use multi-constellation global navigation satellite system (GNSS) signals to achieve the highest possible quality, reliability, and accuracy [1,2]. The first fully operable GNSS system was the American GPS (Global Positioning System) in operation since the 1990s, and the second was the Russian GLONASS, operational since 2011 [3]. The Chinese BeiDou system has also reached full operational capability (FOC) [4]. Currently, only Galileo does not have the planned number of operational satellites in orbit and is entering the FOC phase. Thus, there is relatively less coverage of the BeiDou and Galileo data compared to GPS and GLONASS data [5]. The MGEX (Multi-GNSS Experiment) was established by the IGS (International GNSS Service) in 2012 for tracking, analysing, and providing data from all available GNSS signals [6]. It includes signals from GPS, GLONASS, Galileo, and BeiDou as well as the QZSS (Quasi-zenith Satellite System), NAVIC (Indian Regional Navigational Satellite System), and SBAS (Satellite-based Augmentation System) [7,8]. The MGEX provides observations, clock and orbit files, DCBs (Differential Clock Biases), and broadcast ephemeris [9,10]. In recent years, multiple studies concerning the analysis of clock or orbit products of four GNSS systems have been conducted, e.g., by analysing rapid products [11], considering the correction of inter-satellite code biases [12], prediction models of GNSS satellite clock errors [13], and assessment of multi-GNSS precise orbit and clock products from different analysis centres [14]. Other authors also analysed different aspects using raw clock products from MGEX, like clock estimation using the dual-thread parallel method [15], or by methods of removing day-boundary discontinuities on GNSS clocks [16] or impact of satellite clock offset on differential code biases estimation using undifferenced GPS triple-frequency observations [17]. Adopted in this paper methodology based on outliers removal using MAD (median absolute deviation method) is commonly known, accepted and commonly used, mainly in studies dealing with atomic clocks for removal outliers [18,19,20,21].

In this paper, the authors analysed 8 years (2014–2021) of precise clock products provided by MGEX regarding their availability, quality, and magnitude of the outliers for four GNSS systems: GPS, GLONASS, BeiDou, and Galileo. For this purpose, clock corrections with a 5 min sampling interval were extracted for each of the four available GNSS systems during 2014–2021. For each satellite, their availability during 2014–2021 and the percentage of the outliers were calculated, and the characteristics of clock correction and its frequency before and after outlier removal were determined. In the last part of this paper, a Hadamard deviation (HDEV) was calculated for each year to determine the stability of each clock, its noise, and its changes over time. There are very few publications on this type of research. The clocks with the highest stability and reliability in PPP positioning can be considered reference clocks [22,23]. Furthermore, the uniqueness of the research lies in the use of a long, 8-year analysis period with a very large measurement interval (300 s), which is a novelty among other publications dealing with this topic.

## 2. Methods

In this paper, the authors analysed the full coverage of the clock product data, since this is provided by MGEX from 2014 (1773 GPS weeks) through the end of 2021 (2190 GPS weeks). Files were created by the Center for Orbit Determination in Europe (CODE), as this institution provides the longest and, most importantly, continuous product availability from all of the ACs (analysis centres). Over these 8 years, 120 satellites were analysed: 37 BeiDou (C), 26 Galileo (E), 32 GPS (G), and 25 GLONASS (R).

**Figure 1:**
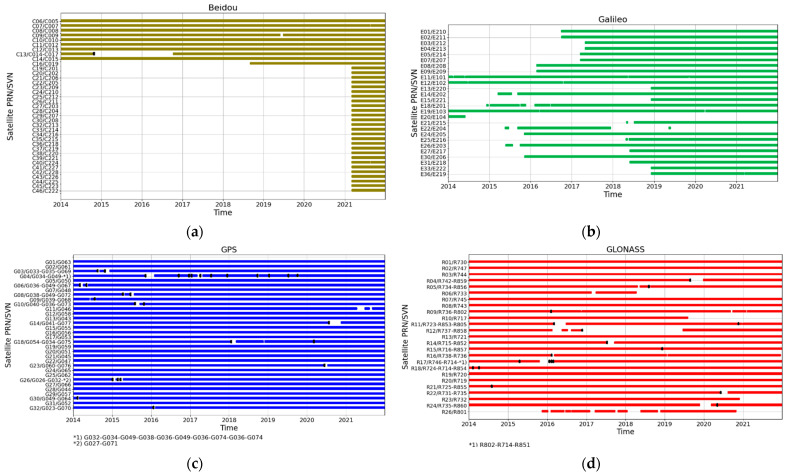
Data coverage of the analysed period for each system and satellite: (**a**) BeiDou; (**b**) Galileo; (**c**) GPS; (**d**) GLONASS. Black dots represent a change in SVN.

Figure 1 shows the time series of the data coverage for each satellite during the period of 2014–2021 (1773-2190 GPS week), where the y-axis represents satellite PRN (Pseudorandom Noise) and SVN (Satellite Vehicle Number) numbers. In our study, the satellite’s PRN number held greater significance, as MGEX’s clock files provide information specific to each satellite’s PRN number. During the analysed time period, the majority of the available data concerned the GPS and GLONASS systems. BeiDou and Galileo are the newest systems and were expanding and sending new satellites during the analysed period. For further analysis, only some of all of the available satellites were chosen. Regarding BeiDou, a majority of the satellites were sent into orbit in 2018, similar to the Galileo system, where a major part of the space segment was sent in the years 2015–2018.

**Figure 2:**
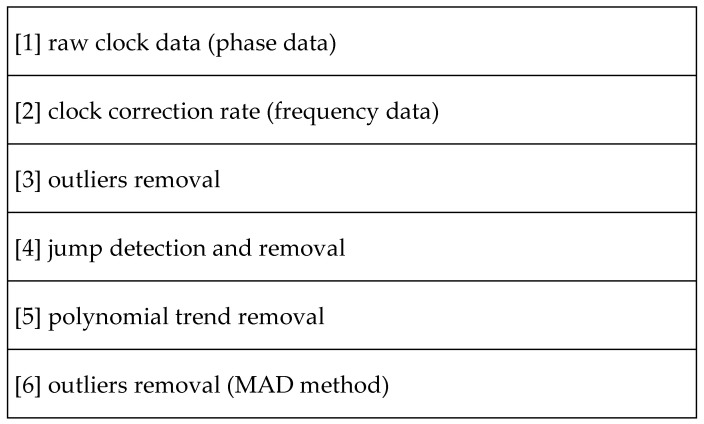
Description of the data processing steps.

The below diagram (Figure 2) is adopted by the authors’ methodology, which is common in similar research [24,25,26].

The number of existing outliers in final clock products for each satellite and for each year was found. Outliers were removed using the MAD (median absolute deviation) method, and a criterion of 3 × MAD was adopted [27,28]. The outlier (jump) is defined and removed by a MAD function. Using the MAD method, 3.4% of data for GPS, 3.7% for GLONASS, 2.7% for Beidou, and 18.9% for GALILEO were removed.

Finally, for each year and satellite, an HDEV (Hadamard Deviation) was calculated to examine the stability of the clocks and their noise. Such a methodology allows for a calculation of the repeatability of stability on an annual basis. For the data analysis, only satellites with at least 50% available data during the analysed period were chosen, which left 81 out of the 120 available satellites: 9 BeiDou, 16 Galileo, 32 GPS, and 24 GLONASS satellites.

## 3. Results

**Figure 3:**
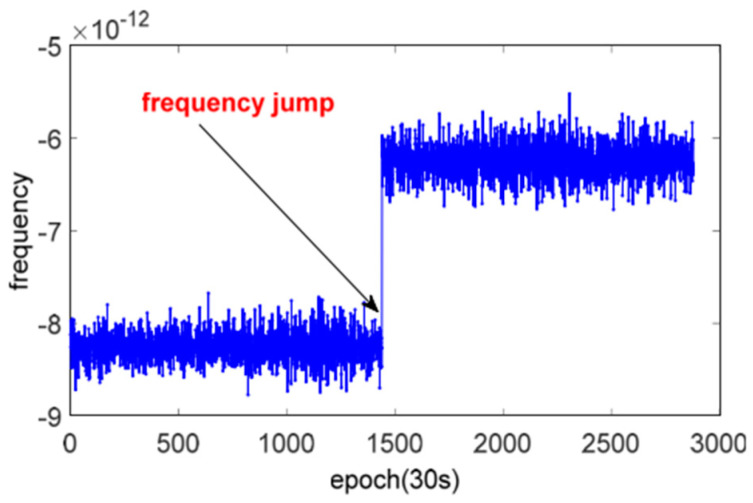
Sample frequency jump of satellite clock.

In the first part of this study, a quality check of 81 analysed satellite clocks was performed and the number of outliers in the clock correction time series was detected using the MAD method described in the previous section. Outlier detection was performed using the frequency domain; a sample outlier is shown in Figure 3. After outlier detection, if a data point was outside of the selected MAD width criterion, it was removed from further analysis. Figure 3 shows a sample raw frequency data of a clock correction file with a relatively big (~2 ps) magnitude, which is eliminated using the MAD method [29].

**Figure 4:**
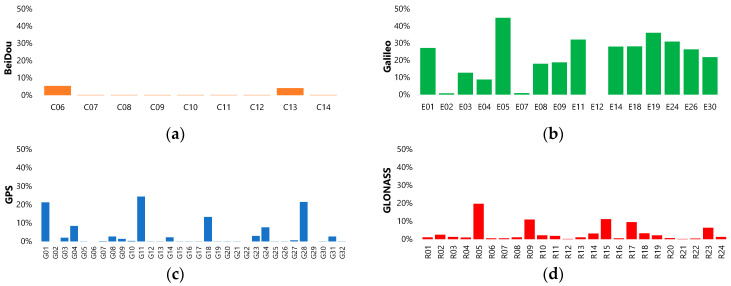
Number of outliers in each set of clock corrections in each satellite divided by GNSS system: (**a**) BeiDou; (**b**) Galileo; (**c**) GPS; (**d**) GLONASS.

A statistical summary of the quality of the analysed data is shown in Figure 4. The above-mentioned satellites show the percentage of outliers for the whole period. For a better comparison of the outliers between GNSS systems, the scale for the y-axis is the same for all charts. The smallest percentage of outliers occurred in the case of the BeiDou satellites, but it should be noted that there were only nine satellites in the analysis. The Galileo system has the biggest percentage of outliers, exceeding 40%. Moreover, the majority of Galileo satellites deliver more than 20% outliers. The GPS and GLONASS systems have very similar results—a small number, <3%—of outliers deleted and a couple of satellites with more than 10% of outliers in clock correction data. No correlation was found between the occurrence of outlying observations and the type of satellite block or type of clock. The phenomenon is purely stochastic in nature.

**Figure 5:**
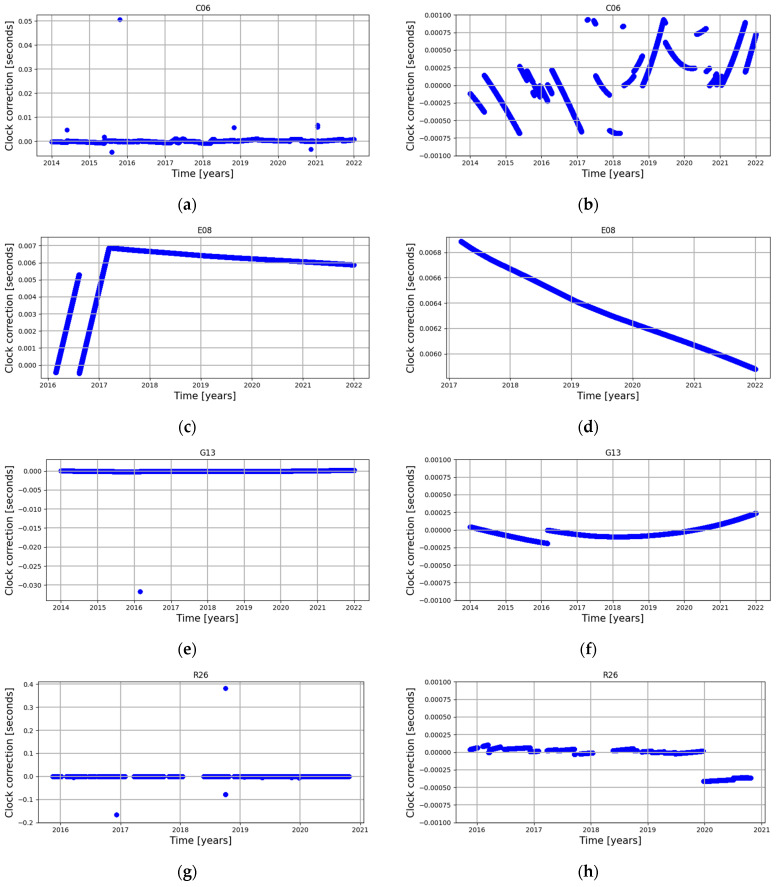
Sample phase graphs: (**a**) satellite C06 raw; (**b**) satellite C06 cleaned; (**c**) satellite E08 raw; (**d**) satellite E08 cleaned; (**e**) satellite G13 raw; (**f**) satellite G13 cleaned; (**g**) satellite R26 raw; (**h**) satellite R26 cleaned.

Figure 5 shows sample satellite clock corrections (phase) for each of the analysed GNSS systems. The left side of Figure 5 shows raw data, while the right shows data cleaned by the MAD method. This shows the different nature of the magnitude, number of outliers and trends in graphs. Removal of the outliers reveals various characteristics of the clock corrections, especially in the case of BeiDou (e.g., C06) and GPS (e.g., G13) satellites. For some satellites, the changing nature of the correction over time is clearly visible, which is due to the corrections made by the control segment; this is particularly evident in the case of satellites C06 and R26. In the case of the E08 satellite, a change in the nature of the correction is visible twice, and in the case of the G13 satellite, once.

**Figure 6:**
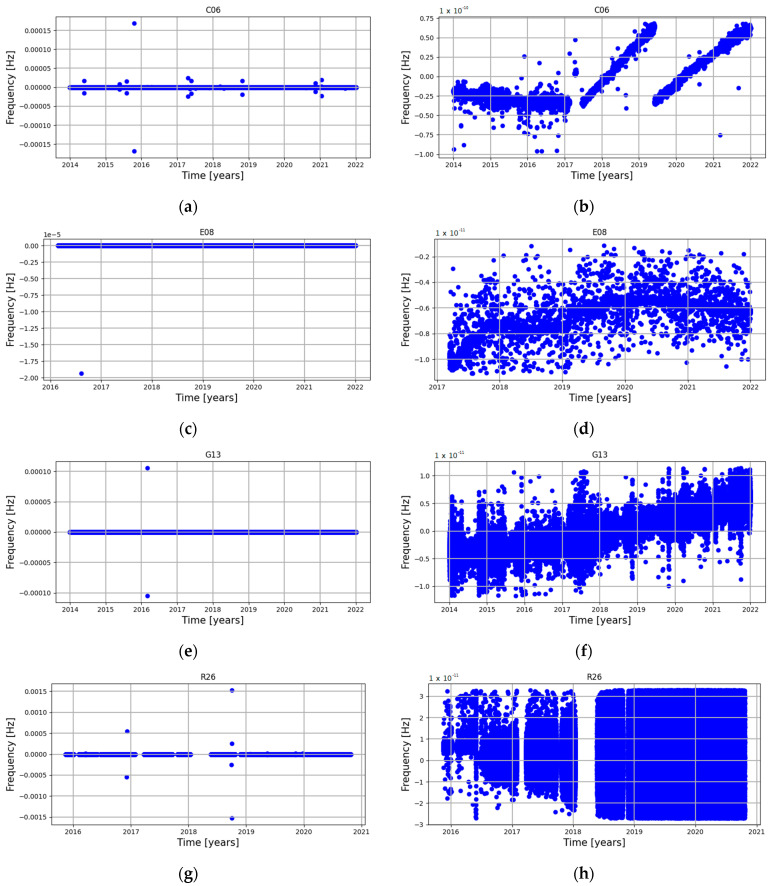
Sample frequency graphs: (**a**) satellite C06 raw; (**b**) satellite C06 cleaned; (**c**) satellite E08 raw; (**d**) satellite E08 cleaned; (**e**) satellite G13 raw; (**f**) satellite G13 cleaned; (**g**) satellite R26 raw; (**h**) satellite R26 cleaned.

Analysis of the satellite clock correction frequency, which is the first derivative of the phase (clock correction), shows a quite different nature; the same sample satellites as in Figure 5 are shown in Figure 6. Firstly, the number and magnitude of the frequency outliers are quite different for each GNSS system. The biggest number of outliers are in the BeiDou and GLONASS systems, which is the result of the many changes of the phase character, the same conclusions, but the magnitude of outliers is greatest in general for the GPS and Galileo systems, followed by the BeiDou and GLONASS systems. Only in the case of the BeiDou satellites is there a visible trend in the frequency, which in the case of C06 changed three times. For other GNSS systems, trends have a stable, non-changeable character, which might be affected only by drift.

**Figure 7:**
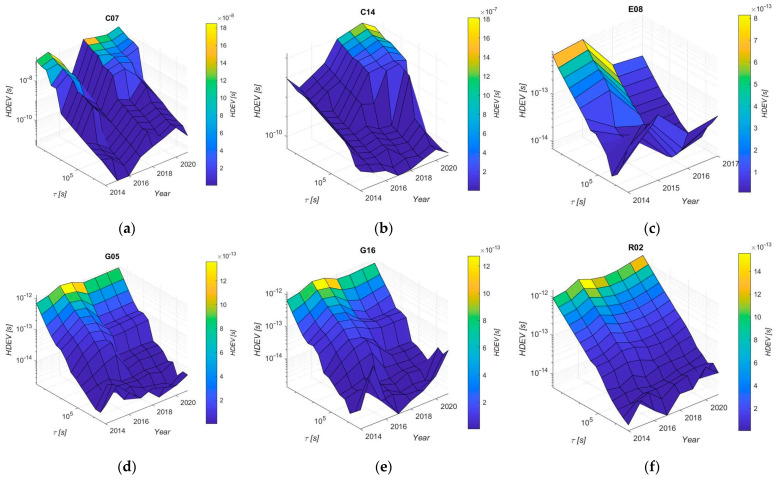
A yearly HDEV of selected satellites: (**a**) satellite C07; (**b**) satellite C14; (**c**) satellite E08; (**d**) satellite G05; (**e**) satellite G16; (**f**) satellite R02.

Figure 7 shows the calculated HDEV for some of the selected satellites, divided into yearly periods. This analysis shows that the number and, more importantly, the magnitude of outliers determines stability. For some satellites, the charts presented below will be incomplete due to them operating for only a part of the year, or because of removed outlying observations. The GPS and GLONASS satellites are the most stable and repeatable according to the HDEV analysis. Comparison of year-to-year HDEV reveals only negligible changes, and stability is as repeatable as possible.

Thus, the repeatability of the clock corrections of the newest systems (BeiDou and Galileo) is now the same as for the other two systems. In the case of sample satellites C07 and C14, changes are clearly visible when comparing year to year. Moreover, Galileo is characterised by the biggest number of outliers of all systems, where the number of satellite clock corrections includes more than 20% of outliers.

## 4. Discussion

Analysis of the outliers results shows that the number of outliers varies depending on the satellite system. The Galileo system has the highest percentage of outliers (~20% on average), which might be an effect of the new system (new satellites, new algorithms, etc.) and, additionally, the high number of outliers is an effect of the trend of the phase graph (Figure 5d). In the case of the other three systems, there are single satellites with a percentage of outliers higher than 3%. For these three systems, we did not find any correlation between the number of outliers and oscillator type or satellite block, so there are no GNSS-specific or block-type-specific phenomena. This phenomenon is only attributed to individual satellites. A comparison of year-to-year stability shows that the stability of the GPS and GLONASS systems is better than the BeiDou and Galileo systems, which means that the repeatability of the clock correction is more consistent.

## 5. Conclusions

In this paper, the authors analysed the longest currently available time series of clock corrections of four GNSS systems—GPS, GLONASS, Galileo and BeiDou. In the last 10 years, the number of Galileo and BeiDou satellites has increased significantly, and these systems are becoming fully operational. GPS and GLONASS signals were available for all satellites, while the Beidou and Galileo satellite segments were still being updated and it is only in the last few years that their constellation has been complete. The standard method used for eliminating outliers (MAD—Median Absolute Deviation) proved to be as sufficient as possible. Conclusions from the analyses performed indicate a very high outlier rate for almost all Galileo satellites, which cannot be said for the other three systems. This indicates the need for further research in this area. As far as stability analysis is concerned, the GPS and GLONASS systems appear to be the most accurate, which is most likely due to the relatively long period of their operation (the first satellites of both systems were launched in the late 1970s and early 1980s), which is indicative of the successive improvements in satellite blocks. This cannot be said of the Galileo and Beidou satellites. These are new systems, still being tested, for which a higher stability comparable to the other two systems will most likely be achieved in the next few years, and it is therefore necessary to continue studying their stability.

## Data Availability

Data are contained within the article.

## References

[1] Specht M. (2022). Experimental Studies on the Relationship Between Hdop and Position Error in the Gps System. Metrol. Meas. Syst..

[2] Magiera W., Vārna I., Mitrofanovs I., Silabrieds G., Krawczyk A., Skorupa B., Apollo M., Maciuk K. (2022). Accuracy of Code GNSS Receivers under Various Conditions. Remote Sens..

[3] Leick A., Rapoport L., Tatarnikov D. (2015). GPS Satellite Surveying.

[4] Brach M. (2022). Rapid Static Positioning Using a Four System GNSS Receivers in the Forest Environment. Forests.

[5] Zhou W., Cai H., Chen G., Jiao W., He Q., Yang Y. (2022). Multi-GNSS Combined Orbit and Clock Solutions at iGMAS. Sensors.

[6] Prange L., Villiger A., Sidorov D., Schaer S., Beutler G., Dach R., Jäggi A. (2020). Overview of CODE’s MGEX solution with the focus on Galileo. Adv. Space Res..

[7] Montenbruck O., Steigenberger P., Prange L., Deng Z., Zhao Q., Perosanz F., Romero I., Noll C., Stürze A., Weber G. (2017). The Multi-GNSS Experiment (MGEX) of the International GNSS Service (IGS)—Achievements, prospects and challenges. Adv. Space Res..

[8] Zrinjski M., Matika K., Barković Đ. (2019). Razvoj i modernizacija GNSS-a. Geod. List.

[9] Montenbruck O., Steigenberger P., Khachikyan R., Weber G., Langley R.B., Mervart L., Hugentobler U. (2014). IGS-MGEX Preparing the Ground for Multi-COnstellation GNSS Science. Inside GNSS.

[10] Maciuk K. (2022). Aging of ground Global Navigation Satellite System oscillators. Eksploat. Niezawodn..

[11] Chen Q., Song S., Zhou W. (2021). Accuracy analysis of gnss hourly ultra-rapid orbit and clock products from shao ac of igmas. Remote Sens..

[12] Chen L., Li M., Zhao Y., Hu Z., Zheng F., Shi C. (2021). Multi-GNSS real-time precise clock estimation considering the correction of inter-satellite code biases. GPS Solut..

[13] Ge H., Li B., Wu T., Jiang S. (2021). Prediction models of GNSS satellite clock errors: Evaluation and application in PPP. Adv. Space Res..

[14] Li W. (2021). Assessment of multi-GNSS precise orbit and clock products from different analysis centers based on precise point positioning. Acta Geodyn. Geomater..

[15] Dai Z., Dai X., Zhao Q., Bao Z., Li C. (2018). Multi-GNSS real-time clock estimation using the dual-thread parallel method. Adv. Space Res..

[16] Rovira-Garcia A., Juan J.M., Sanz J., González-Casado G., Ventura-Traveset J., Cacciapuoti L., Schoenemann E. (2021). Removing day-boundary discontinuities on GNSS clock estimates: Methodology and results. GPS Solut..

[17] Fan L., Shi C., Wang C., Guo S., Wang Z., Jing G. (2020). Impact of satellite clock offset on differential code biases estimation using undifferenced GPS triple-frequency observations. GPS Solut..

[18] Riley W.J. (2008). Handbook of Frequency Stability Analysis.

[19] Jiang N., Xu T., Xu Y., Xu G., Schuh H. (2020). Detecting and repairing inter-system bias jumps with satellite clock preprocessing. Remote Sens..

[20] Jia X., Zeng T., Ruan R., Mao Y., Xiao G. (2019). Atomic clock performance assessment of BeiDou-3 basic system with the noise analysis of orbit determination and time synchronization. Remote Sens..

[21] Li X., Yuan Y., Huang J., Zhu Y., Wu J., Xiong Y., Li X., Zhang K. (2019). Galileo and QZSS precise orbit and clock determination using new satellite metadata. J. Geod..

[22] Nistor S., Buda A.S. (2016). The Influence of Zenith Tropospheric Delay on PPP-RTK. J. Appl. Eng. Sci..

[23] Nistor S., Buda A.S. (2017). Evaluation of the ambiguity resolution and data products from different analysis centers on zenith wet delay using PPP method. Acta Geodyn. Geomater..

[24] He X., Montillet J.P., Hua X., Yu K., Jiang W., Zhou F. (2017). Noise analysis for environmental loading effect on GPS position time series. Acta Geodyn. Geomater..

[25] He L., Zhou H., Zhu S., Zeng P. (2020). An improved QZSS satellite clock offsets prediction based on the extreme learning machine method. IEEE Access.

[26] Xu B., Wang L., Fu W., Chen R., Li T., Zhang X. (2019). A practical adaptive clock offset prediction model for the BeiDou-2 system. Remote Sens..

[27] Lee Y.K., Yang S., Lee H.S., Lee J.K., Lee J.H., Hwang S., Lee J.K., Lee J.H. (2020). Outlier Detection Method for Time Synchronization. J. Position. Navig. Timing.

[28] Cao Y., Huang G., Xie W., Xie S., Wang H. (2021). Assessment and comparison of satellite clock offset between BeiDou-3 and other GNSSs. Acta Geod. Geophys..

[29] Ai Q., Maciuk K., Lewińska P., Borowski Ł. (2021). Characteristics of Onefold Clocks of GPS, Galileo, BeiDou and GLONASS Systems. Sensors.

